# Associations between gut microbiota and adverse neurodevelopmental outcomes in preterm infants: a two-sample Mendelian randomization study

**DOI:** 10.3389/fnins.2024.1344125

**Published:** 2024-02-14

**Authors:** Yuqian Wang, Tongfei Cheng, Yifan Cui, Danyang Qu, Xin Peng, Liu Yang, Xuwu Xiao

**Affiliations:** ^1^Department of Graduate, Dalian Medical University, Dalian, Liaoning, China; ^2^Department of Pediatrics, The Second Hospital of Dalian Medical University, Dalian, Liaoning, China; ^3^Department of Pediatrics, The Affiliated Women’s and Children’s Hospital of Qingdao University, Qingdao, Shandong, China; ^4^Department of Pediatrics, Dalian Women and Children’s Medical Group, Dalian, Liaoning, China

**Keywords:** Mendelian randomization, instrumental variable, gut microbiota, neurodevelopmental outcome, preterm infant, causal relationship, genome-wide association study polymorphism, single nucleotide gastrointestinal microbiome

## Abstract

Gut microbiota are associated with adverse neurodevelopmental outcomes in preterm infants; however, the precise causal relationship remains unclear. In this study, we conducted a two-sample Mendelian randomization (MR) analysis to comprehensively study the relationship between gut microbiota and adverse neurodevelopmental outcomes in preterm infants and identify specific causal bacteria that may be associated with the occurrence and development of adverse neurodevelopmental outcomes in preterm infants. The genome-wide association analysis (GWAS) of the MiBioGen biogroup was used as the exposure data. The GWAS of six common adverse neurodevelopmental outcomes in premature infants from the FinnGen consortium R9 was used as the outcome data. Genetic variations, namely, single nucleotide polymorphisms (SNPs) below the locus-wide significance level (1 × 10^−5^) and genome-wide statistical significance threshold (5 × 10^−8^) were selected as instrumental variables (IVs). MR studies use inverse variance weighting (IVW) as the main method. To supplement this, we also applied three additional MR methods: MR-Egger, weighted median, and weighted mode. In addition, the Cochrane’s Q test, MR-Egger intercept test, Mendelian randomization pleiotropy residual sum and outlier (MR-PRESSO), and leave-one-out methods were used for sensitivity analysis. Our study shows a causal relationship between specific gut microbiota and neurodevelopmental outcomes in preterm infants. These findings provide new insights into the mechanism by which gut microbiota may mediate adverse neurodevelopmental outcomes in preterm infants.

## Introduction

With the gradual improvement in the level of neonatal treatment, the survival rate of preterm infants at small gestational age has been greatly improved; however, the long-term prognosis of the status of their nervous system remains uncertain. Premature infants are born during a critical stage of rapid brain development, which involves intricate and precise programmed neurodevelopmental processes (Lu and Claud, 2019; Parker et al., 2023). However, this delicate period leaves the developing brain vulnerable to various injuries, resulting in a heightened risk of long-term neurocognitive, behavioral, and motor impairments. Factors such as abnormal sensory experiences, toxic stress, systemic inflammation, and early alterations in the infant microbiome have been linked to neurodevelopmental alterations (Aarnoudse-Moens et al., 2009; Patra et al., 2017; Ottolini et al., 2020). These adverse effects on the nervous system of infants pose a significant social and economic burden. Hence, there is a need to identify potential risk factors that could cause various unfavorable neurodevelopmental outcomes in preterm infants.

Some interesting data have emerged on the correlation between early gut microbiota colonization and short-and long-term clinical outcomes in preterm infants (Moschopoulos et al., 2018; Niemarkt et al., 2019; Lee et al., 2020). The neurodevelopment outcomes of infants are affected by various factors. Recent studies have focused on the bilateral interchange between the central nervous system and the gastrointestinal tract (Lu and Claud, 2019). The gut microbiota comprise a dynamic and complex ecological microbial community (O'Hara and Shanahan, 2006). The microbiota and the central nervous system may communicate with each other through the microbiota–gut–brain (MGB) axis, which includes a variety of pathways, including immune responses, vagus and enteric nerves, and molecules or metabolites produced by microorganisms (Cryan et al., 2019). The correlation between changes in the composition of gut microbiota and early brain function has been demonstrated in germ-free mice (GF) that exhibited altered stress, anxiety responses, and memory dysfunction (Diaz et al., 2011; Gareau et al., 2011; Diaz, 2016). Recent studies have shown that the gut microbiota–immune–brain axis may play a role in brain injury in very preterm infants (Seki et al., 2021) and is associated with neurodevelopment at 2 years of age (Roze et al., 2020). The destruction of the microbiome in early life can lead to extensive behavioral and neurological changes during and after development. However, the causal relationship between gut microbiota and neurodevelopmental outcomes in preterm infants remains unclear.

Mendelian randomization (MR) is a method of integrating the summary data of genome-wide association studies (GWAS), similar to randomized controlled trials, using allele randomization during meiosis and subsequent irreversible exposure to the genotype at conception (Burgess et al., 2017; Swanson et al., 2017; Bowden and Holmes, 2019). Using genetic variation as an instrumental variable (IV), MR design is usually less susceptible to residual confounding factors and reverse causality than conventional observational analysis, thereby enhancing the causal relationship between exposure and outcome (Burgess and Thompson, 2017; Chen C. et al., 2020; Chen J. et al., 2020).

Due to the characteristics of Mendelian randomization, we applied a systematic two-sample MR analysis to comprehensively explore whether gut microbiota has causal effects on various neurodevelopmental outcomes in preterm infants and determine specific causal bacterial classifications. By the MR study, we aimed to elucidate the role of gut microbiota in the neurodevelopment of preterm infants to help develop new prevention and treatment strategies, such as probiotic therapy and fecal microbiota transplantation, providing a theoretical basis for brain protection strategies in preterm infants in the future.

## Materials and methods

### Data sources

The GWAS summary level data of gut microbiota were obtained from the MiBioGen study (Kurilshikov et al., 2021). This was the largest, multiethnic, genome-wide meta-analysis of gut microbiota to date, analyzing genome-wide genotyping data and 16S fecal microbiota data from 24 cohorts (18,340 people). Most respondents were of European descent (*N* = 13,266). The V4, V3–V4 and V1–V2 regions of the 16S rRNA gene were used for the classification of microbial composition. Subsequently, direct taxonomy was used for taxonomic classification. After 16S microbiome data processing, 211 taxa were identified, involving 131 genera, 35 families, 20 orders, 16 classes, and 9 phyla.

The GWAS pooled data of cerebral palsy (482 cases, 373,780 controls), intellectual disability (1,136 cases, 277,468 controls), autism (564 cases, 277,526 controls), anxiety (40,191 cases, 277,526 controls), attention deficit hyperactivity disorder (2,340 cases, 371,117 controls), and behavioral and emotional disorders (6,160 cases, 371,117 controls) were obtained from the FinnGen Alliance R9. Detailed data on cohorts, genotypes, endpoint definitions, and association tests in the FinnGen Alliance study are available on the FinnGen website. Table 1 shows the summary data sources and the details of the outcome analyzed in this MR study.

**Table 1:** The summary data sources and details of the outcome.

Outcome					
Trait	Consortium	Samples	Case	Control	Phenocode
Intellectual disability	FinnGen(R9)	278,608	1,136	277,468	KRA_PSY_MENTALRET_EXMORE
CP	FinnGen(R9)	374,262	482	373,780	G6_CP
ADHD	FinnGen(R9)	373,457	2,340	371,117	F5_HYPERKIN
Autism	FinnGen(R9)	278,090	564	277,526	KRA_PSY_AUTISM_EXMORE
Anxiety neurosis	FinnGen(R9)	317,717	40,191	277,526	KRA_PSY_ANXIETY_EXMORE
Behavioral and emotional disorders	FinnGen(R9)	377,277	6,160	371,117	F5_BEHEMOCHILD

### Selection of instrumental variables

**Figure 1:**
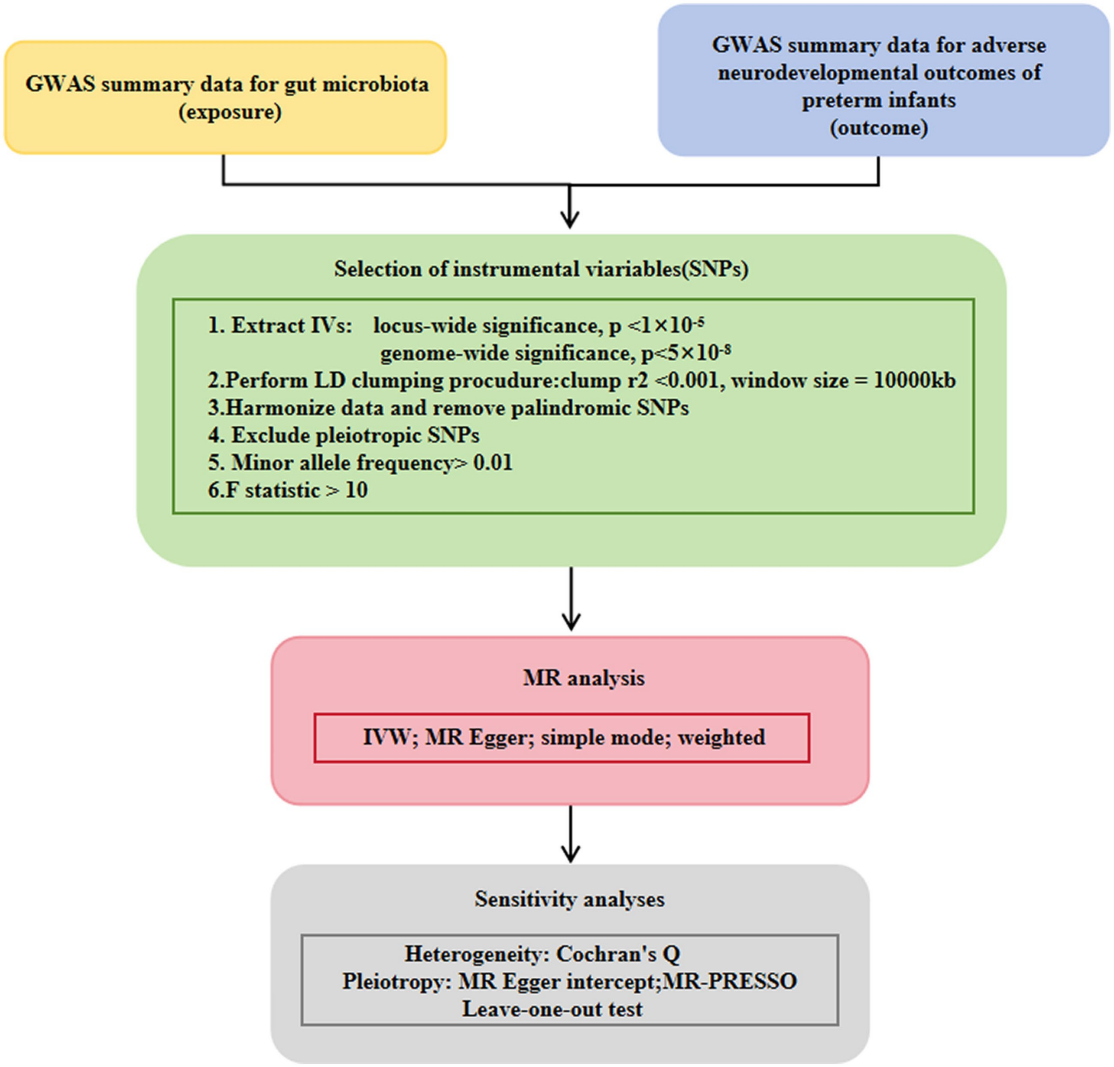
Study design of the present MR study on the associations of gut microbiota and adverse neurodevelopmental outcomes in preterm infants. A flowchart of the whole MR analysis was displayed in this figure. MR, Mendelian randomization; GWAS, genome-wide association study; SNPs, single nucleotide polymorphisms; IVW, inverse-variance weighted; LD, linkage disequilibrium; MR-PRESSO, MR pleiotropy residual sum and outlier.

The flow chart of the study is shown in Figure 1. Briefly, gut microbes were considered as exposures, whereas adverse neurodevelopmental outcomes in preterm infants were considered as outcomes. Bacterial taxa were classified and analyzed at five taxonomic levels (phylum, class, order, family, and genus).

To ensure the authenticity and accuracy of the causal relationship between the intestinal microbiome group and risk of adverse neurodevelopmental outcomes in premature infants, the following quality control steps were used to select the best instrumental variables. Single nucleotide polymorphisms (SNPs) that were significantly associated with the gut microbiota were selected as IVs. Two strategies of MR analyses were conducted. A group of SNPs with genome-wide significance (*p* < 5 × 10^−8^) were selected as IVs. To obtain more comprehensive results and increase the explained phenotypic variance, we selected another group of SNPs below the significance level of the whole locus (*p* < 1 × 10^−5^) as IVs. The minor allele frequency (MAF) threshold of the variation of interest was 0.01. One of the principles of the MR method is the lack of link imbalance (LD) between the included instrumental variables, because the presence of strong LD may lead to result bias. In this study, we used the aggregation process (*r*^2^ < 0.001, aggregation distance = 10,000 kb) to evaluate the LD between the included SNPs. An important step of MR was to ensure that the effect of SNPs on exposure corresponded to the same allele as the effect on outcome. According to this principle, palindromic SNPs were not included as IVs. When an SNP associated with exposure was missing in the outcome GWAS, the proxy SNP significantly associated with the variation of interest was selected (*r*^2^ > 0.8).

### Mendelian randomization analysis

**Figure 2:**
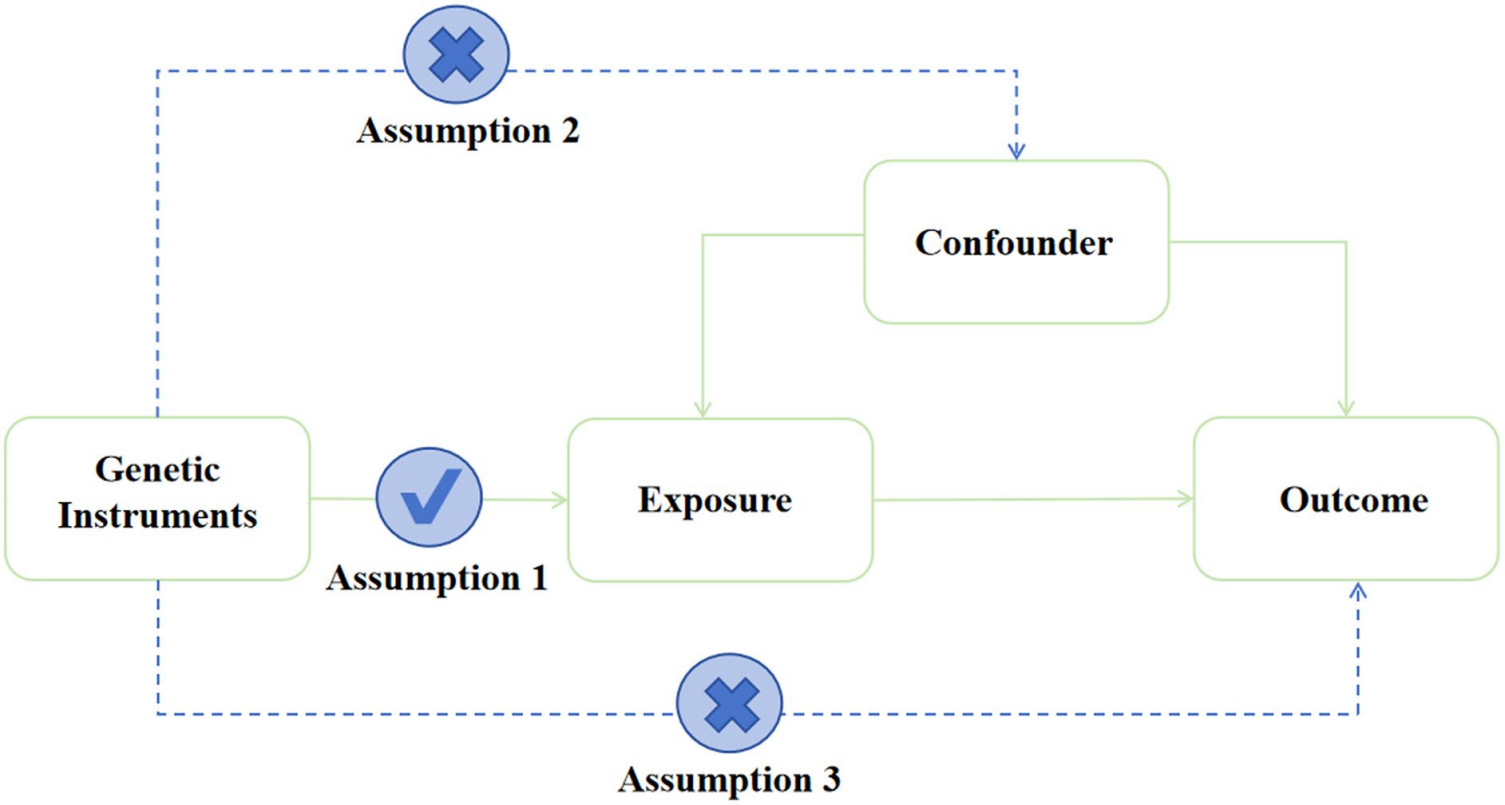
Assumption of the Mendelian randomization study. Directed acyclic graphs for the classical Mendelian randomization designs. The arrows denote causal relations between two variables, pointing from the cause to the effect. The causal pathway is blocked if “X” is placed in the arrowed line.

MR analysis was performed using IVs (Burgess and Thompson, 2011; Davey and Hemani, 2014) and other genetic variations to estimate the causal effect of phenotype on outcomes. MR analysis has three important assumptions, as shown in Figure 2. The first hypothesis is that variation should be associated with exposure. The second is that variation should not be associated with any confounding factors. Finally, the third hypothesis is that the association between genetic variation and outcome can only be achieved through exposure (Burgess and Thompson, 2015).

We used the inverse variance weighting (IVW) method as the main strategy for MR analysis to estimate the causal relationship between gut microbiota composition and neurodevelopmental outcomes in preterm infants (Burgess et al., 2013). To ensure the robustness of the results, we performed three additional MR methods for supplementary analysis (Bowden et al., 2016). Specifically, using the weighted median method, up to half of the instrumental variables are assumed to be invalid to solve the potential strong assumption that deviates from IVW, that is, all instrumental variables are valid. Egger’s regression and MR-PRESSO were used to detect and correct pleiotropic effects and outliers (Bowden et al., 2015; Verbanck et al., 2018; Shrestha, 2019). The leave-one-out analysis was performed to determine whether MR analysis was driven by a single IV. For gut microbiota with more than 3 SNPs, leave-one-out and MR-PRESSO analyses were further performed. The Cochrane’s Q test was also performed to assess the heterogeneity between SNPs associated with each microbial taxon (Shrestha, 2019). F-statistic was used to evaluate the strength of selected IVs and determine whether the estimation of causal association was affected by weak instrumental bias. Weak IVs with F statistic <10 were excluded (Pierce et al., 2011; Hemani et al., 2018). The statistical analyses were performed using R software version 4.3.2. The MR analyses were performed using the TwoSampleMR package and the fastMR package.

## Results

### Selection of instrumental variables

After ensuring quality control, we identified 603, 1852, 623, 1,637, and 6,095 gut microbiota-related SNPs at the phylum, class, order, family, and genus levels, respectively, at a significance level of *p* < 1.0 × 10^−5^. In addition, we identified a total of 184 SNPs that were associated with gut microbiota at the phylum, class, order, family, and genus levels, respectively, at a genome-wide statistical significance level of *p* < 5 × 10^−8^. We further found that the F statistics of IVs significantly related to gut microbiota were all greater than 10, indicating that the estimates were less likely to be affected by weak instrumental bias.

### Locus-wide significance level

The estimates of the IVW test suggested that the genetically predicted relative abundance of 10 bacterial taxa was negatively associated with the risk of intellectual disability, whereas the genetically predicted relative abundance of another 8 bacterial taxa was positively associated with it. Likewise, IVW analyses demonstrated that the genetically predicted relative abundance of 6 bacterial taxa was negatively correlated with the risk of cerebral palsy, whereas the genetically predicted relative abundance of another 7 bacterial taxa was positively associated with it. In addition, the predicted relative abundance of 15 bacterial taxa was negatively correlated with the risk of attention deficit hyperactivity disorder, whereas the predicted relative abundance of another 13 bacterial taxa was positively correlated with it. We observed a tendency toward a protective effect of the genetically predicted abundance of 14 bacterial taxa on autism. On the contrary, the genetically predicted abundance of 21 bacterial taxa was associated with an increased risk of autism. Likewise, we identified a tendency toward a protective effect of the genetically predicted abundance of 23 bacterial taxa on anxiety neurosis. Conversely, the genetically predicted abundance of 14 bacterial taxa was associated with an increased risk of anxiety neurosis. IVW analyses further suggested the causal protective effects of the genetically predicted increased abundance of 20 bacterial taxa on behavioral and emotional disorders. On the contrary, the genetically predicted abundance of 18 bacterial taxa was associated with an increased risk of behavioral and emotional disorders. The detailed statistical results are shown in Supplementary Table S1.

**Figure 3:**
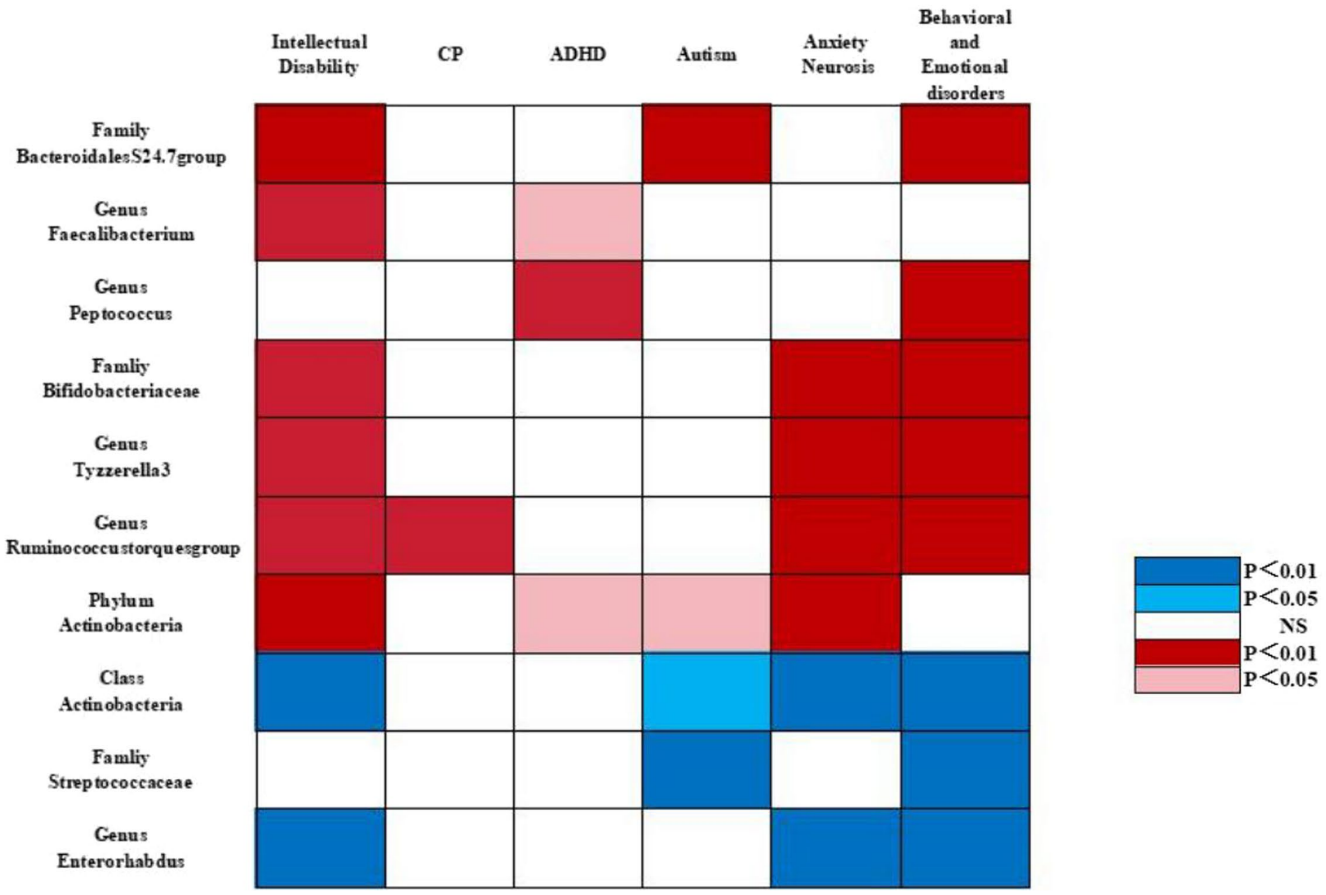
Causal associations of gut microbial genera on neurodevelopmental outcomes in preterm infants identified at the significance from the IVW method. Red represents the protective bacterial traits, blue represents the risk bacterial traits, and white represents no causal bacterial traits. CP, cerebral plasy; ADHD, attention deficit hyperactivity disorder; BED, behavioral and emotional disorders.

**Figure 4:**
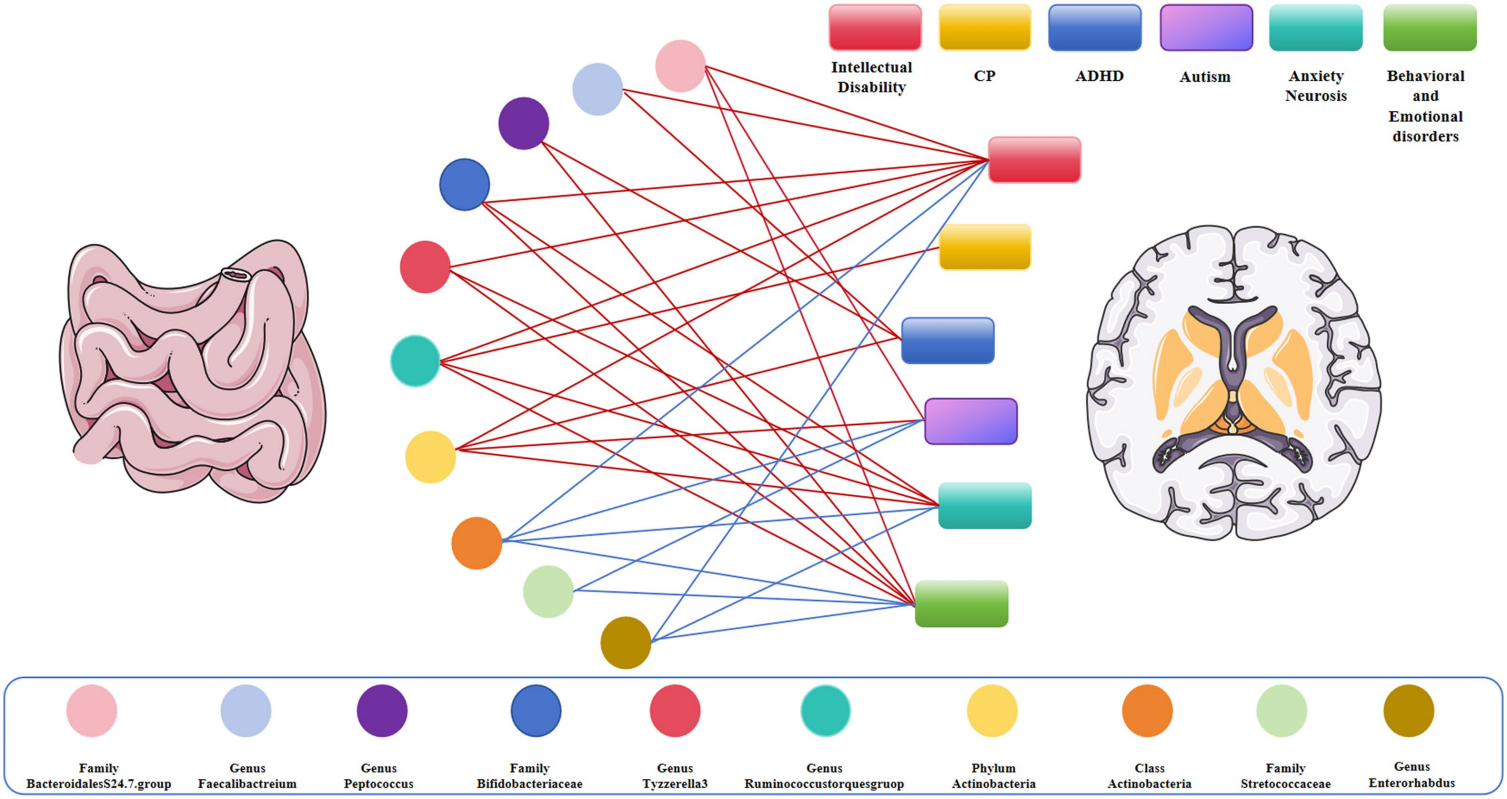
Causal links between gut microbiota and adverse neurodevelopmental outcomes in preterm infants; Red represents the protective link, blue represents the risk link.

In particular, we found that the family Bacteroidales S24.7 group (OR_IVW_ = 0.725, 95% CI = 0.571–0.920, *p* = 0.008) was negatively associated with the risk of intellectual disability. The genus *Faecalibacterium* (OR_IVW_ = 0.650, 95% CI = 0.503–0.840, *p* = 0.001) was negatively associated with the risk of intellectual disability. The family Bacteroidales S24.7 group (OR_IVW_ = 0.500, 95% CI = 0.392–0.638, *p* = 2.52E^−08^) was negatively associated with the risk of autism. The genus *Peptococcus* (OR_IVW_ = 0.869, 95% CI = 0.847–0.892, *p* = 0.001) was negatively correlated with the risk of behavioral and emotional disorders. The family Bacteroidales S24.7 group (OR_IVW_ = 0.878, 95% CI = 0.818–0.942, *p* = 0.0003) was negatively associated with the risk of behavioral and emotional disorders. The genus *Faecalibacterium* (OR_IVW_ = 0.731, 95% CI = 0.564–0.948, *p* = 0.018) and the genus *Peptococcus* (OR_IVW_ = 0.874, 95% CI = 0.840–0.911, *p* = 9.05E^−11^) were negatively correlated with the risk of attention deficit hyperactivity disorder (Supplementary Table S1 and Figures 3, 4).

### Genome-wide statistical significance threshold

The estimates of the IVW test suggested that the genetically predicted relative abundance of 4 bacterial taxa was negatively associated with the risk of intellectual disability, whereas the genetically predicted relative abundance of another 4 bacterial taxa was positively associated with it. We found that the genetically predicted relative abundance of 3 bacterial taxa was negatively correlated with the risk of cerebral palsy, whereas the genetically predicted relative abundance of another bacterial taxon was positively correlated with it. Likewise, IVW analyses suggested causal protective effects of the genetically predicted increased abundance of 2 bacterial taxa on attention deficit hyperactivity disorder risk. We also observed that the genetically predicted relative abundance of only one bacterial taxon was negatively correlated with the risk of autism, whereas the genetically predicted relative abundance of 2 bacterial taxa was positively correlated with it. The genetically predicted abundance of 2 bacterial taxa was associated with an increased risk of anxiety neurosis. By contrast, we detected a tendency toward a protective effect of the genetically predicted abundance of 4 bacterial taxa on anxiety neurosis. Similarly, the protective effect of the genetically predicted abundance of 3 bacterial taxa on behavioral and emotional disorders was counterbalanced by the genetically predicted abundance of another 7 bacterial taxa that were associated with an increased risk of behavioral and emotional disorders.

Interestingly, we identified that Bifidobacteriaceae at the family level (OR_IVW_ = 0.946, 95% CI = 0.924–0.967, *p* = 1.48E^−06^), *Ruminococcus* at the genus level (OR_IVW_ = 0.419, 95% CI, 0.275–0.638, *p* = 5.11E^−05^), Actinobacteria at the phylum level (OR_IVW_ = 0.661, 95% CI = 0.543–0.805, *p* = 5.11E^−05^), and *Tyzzerella-3* at the genus level (OR_IVW_ = 0.743, 95% CI = 0.642–0.859, *p* = 6.17E^−05^) were negatively associated with the risk of intellectual disability. Likewise, we observed that *Ruminococcus* at the genus level (OR_IVW_ = 0.356, 95% CI = 0.171–0.740, *p* = 0.005) were negatively correlated with the risk of cerebral plasy, while Actinobacteria at the phylum level were negatively correlated with the risk of attention deficit hyperactivity disorder (OR_IVW_ = 0.9878, 95% CI = 0.832–0.926, *p* = 2.20E^−06^) and with the risk of autism (OR_IVW_ = 0.588, 95% CI = 0.378–0.953, *p* = 0.018). Of note, we found that Bifidobacteriaceae at the family level (OR_IVW_ = 0.853, 95% CI = 0.822–0.884, *p* = 7.08E^−18^), *Tyzzerella-3* at the genus level (OR_IVW_ = 0.839, 95% CI = 0.799–0.880, *p* = 4.77E^−09^), *Ruminococcus* at the genus level (OR_IVW_ = 0.810, 95% CI = 0.706–0.931, *p* = 0.002), and Actinobacteria at the phylum level (OR_IVW_ = 0.872, 95% CI = 0.817–0.931, *p* = 3.93E^−05^) were negatively associated with the risk of anxiety neurosis. In addition, Bifidobacteriaceae at the family level (OR_IVW_ = 0.727, 95% CI = 0.677–0.781, *p* = 1.29E^−18^), *Ruminococcus* at the genus level (OR_IVW_ = 0.644, 95% CI = 0.520–0.798, *p* = 5.83E^−05^), and *Tyzzerella-3* at the genus level (OR_IVW_ = 0.747, 95% CI = 0.677–0.823, *p* = 4.77E^−09^) were negatively associated with the risk of behavioral and emotional disorders (Tables 2–5 and Figures 3, 4).

**Table 2:** SNPs used as instrumental variables from gut microbiome and intellectual disability GWASs (*p* < 5 × 10–8).

Classification		Nsnp	Methods	Beta	SE	OR (95%CI)	*p*-value	Test of heterogeneity		Horizontal pleiotropy		
								Q	*p*-value	Egger intercept	SE	*p*-value
Family	Bifidobacteriaceae	1,228	MR Egger	−0.079	0.102	0.923 (0.775, 1.129)	0.436	0.000	1.000	0.002	0.010	0.815
			Weighted median	−0.240	0.016	0.786 (0.761, 0.812)	7.94E-38					
			IVW	−0.056	0.012	0.946 (0.924, 0.967)	1.48E-06	0.000	1.000			
			Weighted mode	−0.383	0.069	0.682 (0.596, 0.781)	3.43E-08					
Genus	Enterorhabdus	25	MR Egger	0.406	0.082	1.444 (1.163, 1.689)	0.019	0.000	1.000	0.001	0.013	0.915
			Weighted median	0.408	0.088	1.504 (1.264, 1.789)	4.23E-06					
			IVW	0.407	0.069	1.502 (1.313, 1.718)	3.25E-09	0.007	1.000			
			Weighted mode	0.407	0.153	1.503 (1.114, 2.028)	0.013					
	Ruminococcustorquesgroup	9	MR Egger	−1.89E-16	9.322	1.000 (0,86123175.177)	1.000	0.000	1.000	−0.052	0.559	0.928
			Weighted median	−0.869	0.215	0.419 (0.275, 0.638)	5.11E-05					
			IVW	−0.871	0.169	0.419 (0.300, 0.583)	2.60E-07	0.009	1.000			
			Weighted mode	−0.861	0.292	0.423 (0.239, 0.748)	0.018					
	Tyzzerella3	10	MR Egger	−0.197	5.193	0.821 (0,21596.688)	0.970	0.006	1.000	−0.013	0.673	0.985
			Weighted median	−0.299	0.087	0.741 (0.624, 0.880)	0.0006					
			IVW	−0.297	0.074	0.743 (0.642, 0.859)	6.17E-05	0.006	1.000			
			Weighted mode	−0.301	0.129	0.740 (0.574, 0.954)	0.045					
Class	Actinobacteria	36	MR Egger	1.250	0.517	3.491 (1.267, 9.621)	0.021	7.094	1.000	−0.099	0.053	0.073
			Weighted median	0.329	0.066	1.391 (1.221, 1.584)	6.72E-07					
			IVW	0.299	0.052	1.350 (1.219, 1.494)	6.85E-09	10.507	1.000			
			Weighted mode	0.327	0.110	1.387 (1.117, 1.722)	0.005					
Phylum	Actinobacteria	14	MR Egger	−4.285	4.843	0.014 (0, 182.640)	0.393	6.014	0.915	0.327	0.409	0.440
			Weighted median	−0.509	0.127	0.601 (0.468, 0.770)	5.85E-05					
			IVW	−0.414	0.101	0.661 (0.543, 0.805)	3.81E-05	6.653	0.919			
			Weighted mode	−0.5097	0.175	0.601 (0.426, 0.847)	0.012					

**Table 3:** SNPs used as instrumental variables from gut microbiome and cerebral plasy, attention deficit hyperactivity disorder, Autism GWASs (*p* < 5 × 10–8).

Classification		Nsnp	Methods	Beta	SE	OR (95%CI)	*p*-value	Test of heterogeneity		Horizontal pleiotropy		
								Q	*p*-value	Egger intercept	SE	*p*-value
Cerebral plasy												
Genus	Ruminococcustorquesgroup	9	MR Egger	2.09E-16	20.566	1.000 (0.000, 321078686601167000.000)	1	0.000	1.000	−0.062	1.233	0.961
			Weighted median	−1.031	0.461	0.357 (0.145, 0.880)	0.025					
			IVW	−1.032	0.373	0.356 (0.171, 0.740)	0.005	0.003	1.000			
			Weighted mode	−1.021	0.640	0.360 (0.103, 1.264)	0.149					

Attention deficit hyperactivity disorder											
Phylum	Actinobacteria	343	MR Egger	0.455	0.278	1.577 (0.914, 2.721)	0.102	74.044	−0.013	0.021	0.235
			Weighted median	0.041	0.037	1.042 (0.969, 1.120)	0.034				
			IVW	−0.130	0.027	0.878 (0.832, 0.926)	2.20E-06	78.526			
			Weighted mode	0.044	0.094	1.045 (0.870, 1.257)	0.637				

Autism											
Phylum	Actinobacteria	14	MR Egger	2.919	10.880	18.534 (0, 33840312754.636)	0.793	6.158	−0.292	0.920	0.757
			Weighted median	−0.605	0.284	0.546 (0.313, 0.953)	0.033				
			IVW	−0.530	0.225	0.588 (0.378, 0.915)	0.018	6.259			
			Weighted mode	−0.595	0.376	0.551 (0.264, 1.154)	0.137				
Class	Actinobacteria	36	MR Egger	2.123	1.159	8.359 (0.861, 81.153)	0.075	9.221	−0.194	0.120	0.115
			Weighted median	0.250	0.150	1.284 (0.956, 1.725)	0.046				
			IVW	0.257	0.116	1.294 (1.030, 1.624)	0.026	11.836			
			Weighted mode	0.248	0.246	1.282 (0.791, 2.080)	0.320				

**Table 4:** SNPs used as instrumental variables from gut microbiome and Anxiety neurosis GWASs (*p* < 5 × 10–8).

Classification		Nsnp	Methods	Beta	SE	OR (95%CI)	*p*-value	Test of heterogeneity		Horizontal pleiotropy		
								Q	*p*-value	Egger intercept	SE	*p*-value
Family	Bifidobacteriaceae	30	MR Egger	−0.692	0.216	0.500 (0.327, 0.765)	0.003	15.690	0.970	0.018	0.023	0.070
			Weighted median	−0.177	0.024	0.837 (0.798, 0.879)	8.92E-13					
			IVW	−0.159	0.018	0.853 (0.822, 0.884)	7.08E-18	29.000	0.828			
			Weighted mode	−0.179	0.0393	0.836 (0.770, 0.903)	8.46E-05					
Genus	Enterorhabdus	25	MR Egger	−2.59E-15	3.049	1.000 (0.047, 21.332)	1	0.000	1.000	0.020	0.148	0.891
			Weighted median	0.412	0.060	1.242 (1.173, 1.315)	7.68E-12					
			IVW	0.411	0.044	1.241 (1.187, 1.297)	1.19E-20	0.019	1.000			
			Weighted mode	0.412	0.099	1.241 (1.119, 1.377)	0.0003					
	Ruminococcustorquesgroup	9	MR Egger	1.23E-16	6.03591099	1.000 (0.002, 417.425)	1	0.000	1.000	−0.013	0.185	0.947
			Weighted median	−0.439	0.142	0.811 (0.706, 0.931)	0.002					
			IVW	−0.440	0.109	0.810 (0.726, 0.904)	5.83E-05	0.005	1.000			
			Weighted mode	−0.435	0.196	0.812 (0.662, 0.997)	0.057					
	Tyzzerella3	9	MR Egger	−0.308	1.711	0.735 (0.026, 21.023)	0.862	0.128	1.000	0.017	0.222	0.941
			Weighted median	−0.293	0.063	0.836 (0.786, 0.889)	4.32E-06					
			IVW	−0.292	0.049	0.839 (0.799, 0.880)	4.77E-09	0.134	1.000			
			Weighted mode	−0.294	0.091	0.835 (0.761, 0.916)	0.012					
Class	Actinobacteria	36	MR Egger	0.717	0.334	2.384 (1.158, 1.256)	0.039	35.131	0.414	−0.011	0.018	0.967
			Weighted median	0.360	0.044	1.259 (1.696, 3.352)	4.62E-16					
			IVW	0.315	0.033	1.206 (1.158, 1.256)	3.75E-21	51.178	0.038			
			Weighted mode	0.360946116	0.070875636	1.262 (1.186, 1.343)	1.21E-05					
Phylum	Actinobacteria	14	MR Egger	−2.69	1.602	0.068 (0.003, 1.563)	0.118	5.367	0.945	0.216	0.135	0.136
			Weighted median	−0.178	0.041	0.836 (0.771, 0.907)	1.68E-05					
			IVW	−0.136	0.033	0.872 (0.817, 0.931)	3.93E-05	7.914	0.849			
			Weighted mode	−0.180	0.056	0.835 (0.748, 0.933)	0.007					

**Table 5:** SNPs used as instrumental variables from gut microbiome and behavioral and emotional disordersGWASs (*p* < 5 × 10–8).

Classification		Nsnp	Methods	Beta	SE	OR (95%CI)	*p*-value	Test of heterogeneity		Horizontal pleiotropy		
								Q	*p*-value	Egger intercept	SE	*p*-value
Family	Bifidobacteriaceae	30	MR Egger	−0.922	0.423	0.397 (0.173, 0.911)	0.037	10.202	0.999	0.065	0.046	0.163
			Weighted median	−0.339	0.047	0.712 (0.648, 0.782)	1.15E-12					
			IVW	−0.318	0.036	0.727 (0.677, 0.781)	1.29E-18	12.256	0.997			
			Weighted mode	−0.342	0.078	0.710 (0.608, 0.829)	0.0001					
Class	Actinobacteria	36	MR Egger	0.717	0.334	2.050 (1.064, 3.950)	0.039	17.207	0.993	−0.042	0.035	0.235
			Weighted median	0.360	0.044	1.433 (1.314, 1.564)	4.62E-16					
			IVW	0.315	0.033	1.370 (1.284, 1.463)	3.75E-21	18.668	0.989			
			Weighted mode	0.360	0.070	1.435 (1.249, 1.648)	1.21E-05					
Genus	Ruminococcustorquesgroup	52	MR Egger	1.23E-16	6.035	1.000 (0,137363.400)	1	0.000	1.000	−0.026	0.362	0.944
			Weighted median	−0.439	0.142	0.644 (0.487, 0.852)	0.002					
			IVW	−0.440	0.109	0.644 (0.520, 0.798)	5.83E-05	0.005	1.000			
			Weighted mode	−0.423	0.123	0.723 (0.432, 0.856)	0.011					
	Tyzzerella3	9	MR Egger	−0.291	0.053	0.646 (0.518, 0.735)	0.004	0.000	1.000	−0.038	0.011	0.743
			Weighted median	−0.293	0.063	0.746 (0.658, 0.845)	4.32E-06					
			IVW	−0.292	0.049	0.747 (0.677, 0.823)	4.77E-09	0.006	1.000			
			Weighted mode	−0.294	0.0913	0.745 (0.622, 0.891)	0.012					

By contrast, we identified that Actinobacteria at the class level (OR_IVW_ = 1.350, 95% CI = 1.219–1.494, *p* = 6.85E^−09^) and *Enterorhabdus* at the genus level (OR_IVW_ = 1.502, 95% CI =1.313–1.718, *p* = 0.007) were positively associated with the risk of intellectual disability. We also found that Streptococcaceae at the family level (OR_IVW_ = 1.226, 95% CI = 1.170–1.285, *p* = 1.58E^−17^), Actinobacteria at the class level (OR_IVW_ = 1.370, 95% CI = 1.284–1.463, *p* = 3.75E^−21^), and *Enterorhabdus* at the genus level (OR_IVW_ = 1.509, 95% CI = 1.384–1.645, *p* = 1.19E^−20^) were positively associated with an increased risk of behavioral and emotional disorders. Likewise, Streptococcaceae at the family level (OR_IVW_ = 1.730, 95% CI = 1.163–2.572, *p* = 0.006), and Actinobacteria at the class level (OR_IVW_ = 1.294, 95% CI = 1.030–1.624, *p* = 0.026) were positively correlated with autism risk. Finally, we determined that *Enterorhabdus* at the genus level (OR_IVW_ = 1.241, 95% CI = 1.187–1.297, *p* = 1.19E^−20^) and Actinobacteria at the class level (OR_IVW_ = 1.206, 95% CI = 1.158–1.256, *p* = 3.75E^−21^) were associated with an increased risk of anxiety neurosis, while Actinobacteria at the class level (OR_IVW_ = 1.370, 95% CI = 1.284–1.463, *p* = 3.75E^−21^) were also associated with an increased risk of behavioral and emotional disorders (Tables 2–5 and Figures 3, 4).

### Sensitivity analysis

The results of Cochrane’s Q test revealed partial heterogeneity (Supplementary Table S2). We also did not detect any significant horizontal pleiotropy in the results of MR-Egger intercept analysis and MR-PRESSO global test (Supplementary Tables S3, S4). In addition, the leave-one-out analysis showed that following the removal of any IV, the robustness of the MR results did not change the overall results (Supplementary Figure S1).

## Discussion

In this study, by using large-scale GWAS summary data, we identified a causal relationship between the predicted abundance of genes in specific bacterial groups and six common adverse neurodevelopmental outcomes in preterm infants (cerebral palsy, intellectual disability, anxiety neurosis, autism, behavioral and emotional disorders, attention deficit hyperactivity disorder).

With the continuous improvement in the level of neonatal intensive care, the mortality rate of preterm infants has decreased significantly over time. However, increased infant survival has been associated with a significantly increased risk of severe illness and lifelong neurodevelopmental disorders, such as cerebral palsy, autism spectrum disorders, anxiety, and intellectual disability (Matthews et al., 2018; Pierrat et al., 2021). In the past 20 years, the effects of the gut microbiome on host health and physiological processes, including neural development, have become the subject of increasing research (Clarke et al., 2013; Hsiao et al., 2013; Warner, 2019). Evidence suggests that bacteria in the gut affect brain function (Bresesti et al., 2022). However, only a few studies have explored the relationship between the composition of the gut microbiome and neurodevelopment in preterm infants. Currently, the regulation of the immune system, microbial metabolites, and activation of the vagus nerve are the most studied pathways connecting gut microbiota and neurodevelopment. The effect of preterm infant microbiota on brain development is mediated by local and systemic IGF-1 levels and neuroinflammation (Clarke et al., 2013). As an intestinal microbial metabolite, Butyrate is a HDAC inhibitor. Butyrate can restore histone acetylation and increase the expression of learning-related genes (Moschopoulos et al., 2018).

Late pregnancy refers to the period after 28 weeks of pregnancy, which is a critical period for the development of fetal brain function. During this period, the volume of the brain increases significantly, and cognitive function increases in complexity (Rogers et al., 2016; Matthews et al., 2018). Increasing evidence suggest that in the early stages of life, the gut microbiota is involved in bidirectional signaling between the gut and the brain, forming the so-called microbiota-gut-brain axis (MGBA) (Warner, 2019). The specific intestinal structure and immune immaturity of premature infants, coupled with specific environmental conditions (mode of delivery, neonatal intensive care unit procedures and environment, management, and feeding), can seriously interfere with healthy microbial colonization (La Rosa et al., 2014). In addition, in preterm infants, the relationship between microbial genes and the host may be severely impaired, making preterm infants prone to adverse consequences such as necrotizing enterocolitis (NEC) and late-onset sepsis (LOS), which will eventually interfere with MGBA and affect brain development, resulting in adverse neurodevelopmental outcomes (Underwood et al., 2020).

Compared with healthy full-term newborns, the bacterial diversity of premature infants is lower. Bacteria such as *Enterococcus*, *Escherichia coli*, *Staphylococcus*, *Streptococcus*, and *Clostridium* are common, whereas beneficial strains such as *Bifidobacterium* and *Bacteroides* are colonized relatively late (Butel et al., 2007; Jost et al., 2012; Moles et al., 2013). In premature infants, the diversity of gut microbiota is reduced, exhibiting extensive variation among individuals, and an increase in the proportion of potential pathogens.

Based on existing evidence, only limited clinical studies have been conducted. Monitoring the dynamic changes in the composition of gut microbiota in the first few months of life of premature infants can reveal its possible relationship with later neurodevelopmental outcomes. One significant finding was that the absence of *Bifidobacteria* in preterm infants after 30 d of birth was associated with early neurodevelopmental disorders in children (Oliphant et al., 2021; Beghetti et al., 2022). Conversely, the use of *Bifidobacteria* strains has been shown to promote optimal neurocognitive development in susceptible infants. In addition, in premature infants with unsatisfactory head circumference growth trajectory, a decrease in the abundance of *Bacteroidaceae* and *Lachnomycaceae* (Oliphant et al., 2021). The overgrowth of *Klebsiella* in the intestine is highly predictive of brain injury and has been associated with proinflammatory immune responses (Seki et al., 2021). Furthermore, using a functional log-contrast regression model, researchers identified specific orders (Clostridium, Lactobacillus, and Enterobacter) and genera (*Porphyromonas*, *Enterococcus*, and *Shigella*) in the composition of microbiota that were associated with infant neurobehavioral outcomes as assessed by the Stress/Withdrawal Subscale (NSTRESS) (Sun et al., 2020). This study preliminarily suggested that neonatal intestinal flora plays a role in early cognitive and behavioral neurodevelopment. Various studies have shown that injection of *Lactobacillus acidophilus* and *Bifidobacterium infantis* into pregnant mice promoted brain development and protected the brain of offspring from inflammatory damage after birth (Lu et al., 2020). Some studies have pointed out the potential role of the early colonization by specific bacteria, especially *Bifidobacteria*, in early neurodevelopment in children. Specifically, the absence or low relative abundance of *Bifidobacteria* may constitute a susceptible and immature biomarker, suggesting early intervention strategies during hospitalization and after discharge in neonatal intensive care units to promote optimal neurodevelopment in preterm infants (van den Berg et al., 2016). In addition, *Bifidobacteria* are known to play a pioneering role in the healthy development of infants, contributing to the fine-tuning of the immune system, and possibly exerting neuroprotective effects by regulating the production and release of neuroactive metabolites (Rabe et al., 2020).

In our study, we found that class Actinomycetes showed a positive causal relationship with autism, anxiety neurosis, intellectual disability, behavioral and emotional disorders, family Streptococcaceae had a positive causal relationship with autism, behavioral and emotional disorders, genus *Enterorhabdus* exhibited a positive causal relationship with anxiety disorders and behavioral emotional disorders, and genus *Ruminococcus* had a positive causal relationship with cerebral palsy. Conversely, family Bifidobacteriaceae and genus *Tyzzerella-3* showed a negative causal relationship with intellectual disability, anxiety neurosis, and behavioral and emotional disorders, genus *Peptococcus* had a negative causal relationship with attention deficit hyperactivity disorder and behavioral and emotional disorders, and genus *Faecalibacterium* exhibited a negative causal relationship with intellectual disability and attention deficit hyperactivity disorder. Interestingly, the family Bacteroidales S24.7 group was associated with a protective effect on autism, intellectual disability, and behavioral and emotional disorders. In summary, the family Bifidobacteriaceae and the Bacteroidales S24.7 group were correlated with a protective effect on the neurodevelopment of premature infants, whereas the family Streptococcaceae and class Actinomycetes were found to be dangerous for the neurodevelopmental outcome of premature infants, in consistency with previous findings. Our study revealed a causal relationship between specific gut microbiota and adverse neurodevelopmental outcomes in preterm infants.

The advantages of this study were as follows: First, the MR design reduced residual confusion and reverse causality, thereby improving causal inference in the relationship between the composition of gut microbes and neurodevelopmental outcomes in preterm infants. Second, we comprehensively studied the correlation of gut microbes with six common adverse neurological outcomes in preterm infants. Third, the causal relationship identified in our MR analysis may provide candidate microbial groups for subsequent functional studies and thus contribute to the development of new methods for the prevention and treatment of adverse neurological outcomes in preterm infants by targeting specific intestinal bacteria.

However, our study had some limitations. First, the identified SNPs based on the genome-wide statistical significance threshold (5 × 10^−8^) were limited. Therefore, in this study, we included SNPs that met the full-site significance level (1 × 10^−5^). Second, this study mainly included individuals of European ancestry, so extrapolation of the results to other populations may limited. In addition, the sample size was small due to insufficient studies on this topic. This may have partially affected our findings. Third, the transgene-related GWAS abstracted data set included in this study was based on 16S rRNA sequencing; more advanced methods, such as metagenomic sequencing, are needed in the future to further assess the species level on the basis of large-scale studies. In addition, this study can be substantiated by adding animal experiments in the future.

In summary, our study provides genetic evidence for the causal effect of gut microbiota on adverse neurodevelopmental outcomes in preterm infants. The identification of both beneficial and harmful gut microbiota that are associated with the risk of adverse neurodevelopmental outcomes in preterm infants may provide valuable insights into the pathogenesis of microbiota-mediated adverse neurodevelopment outcomes in preterm infants and guide effective prevention and treatment strategies in the future.

## Data availability statement

The original contributions presented in the study are included in the article/Supplementary material, further inquiries can be directed to the corresponding authors.

## Author contributions

YW: Writing – original draft. TC: Conceptualization, Writing – review & editing. YC: Data curation, Writing – review & editing. DQ: Methodology, Software, Writing – original draft. XP: Investigation, Writing – original draft. LY: Funding acquisition, Writing – review & editing. XX: Writing – review & editing.
